# Fatal Rickettsia conorii subsp. israelensis Infection, Israel

**DOI:** 10.3201/eid1405.071278

**Published:** 2008-05

**Authors:** Miriam Weinberger, Avi Keysary, Judith Sandbank, Ronit Zaidenstein, Avi Itzhaki, Carmela Strenger, Moshe Leitner, Christopher D. Paddock, Marina E. Eremeeva

**Affiliations:** * Assaf Harofeh Medical Center, Zerifin, Israel; † Tel Aviv University, Ramat Aviv, Israel; ‡ Israel Institute for Biological Research, Ness-Ziona, Israel; § Centers for Disease Control and Prevention, Atlanta, Georgia, USA

**Keywords:** PCR, Israel, epidemiology, Rickettsia infections, Rickettsia conorii, genetics, pathogenicity, travel, dispatch

## Abstract

Underdiagnosis of fatal spotted fever may be attributed to nonspecific clinical features and insensitive acute-phase serologic studies. We describe the importance of molecular and immunohistochemical methods in establishing the postmortem diagnosis of locally acquired Israeli spotted fever due to *Rickettsia conorii* subsp. *israelensis* in a traveler returning to Israel from India.

## Abstract 2

Fatal *Rickettsia conorii* subsp. *israelensis* Infection, Israel

***Rickettsia*** *conorii* subspecies *israelensis,* the cause of Israeli spotted fever (ISF), has been described in Israel, Italy, and Portugal. ISF is characterized by fever, headache, and rash after a tick bite (*1**,**2*). Since nonspecific clinical symptoms occur during disease onset, and no eschar is present in most Israeli cases, ISF, like other rickettsioses, may be misdiagnosed. Fatal outcome has been described in previously healthy children and adults, particularly when appropriate and timely antimicrobial drug treatment was not administered (*2*).

Serologic tests are the most widely available diagnostic tools for spotted fever, but they are less than optimal for the diagnosis of rickettsial diseases in the acute phases (*3*). Autopsy findings may be nondiagnostic unless specialized molecular and immunohistochemical techniques or cell culture-based methods are used to detect rickettsiae (*3**–**5*). We report a confirmed case of fatal spotted fever in Israel due to *R. conorii* subsp. *israelensis*.

## The Case

A 51-year-old previously healthy Israeli man was admitted to Assaf Harofeh Medical Center in Israel for febrile illness 1 month after he had returned from a trip to India. The patient lived in an urban environment in Israel and owned a dog. He had made two 1-month long business trips to India in March and in August 2005. He had been vaccinated against hepatitis A and typhoid fever, but had not taken antimalarial prophylaxis.

The patient’s symptoms started on September 23, 2005, with fever as high as 39°C accompanied by headache, weakness, and frequent urination. After he was given cefuroxime sodium, a generalized rash developed. He was then referred to the hospital on September 26. On admission, he was febrile (38.9°C), and a physical examination showed diffuse macular rash on the trunk, extremities, the palms of his hands, and the soles of his feet. An allergy to cephalosporins was suspected and cefuroxime was discontinued. Results of the following studies were nondiagnostic: routine blood and urine cultures; blood smears for malaria parasites; and serologic tests for HIV, hepatitis A, B, and C, West Nile virus, dengue virus, *Leptospira* spp., cytomegalovirus, and Epstein-Barr virus. The patient’s condition worsened; on day 4 of hospitalization, severe muscle pain, tachycardia (189/min), tachypnea (40/min), oliguria, and generalized convulsions had developed. Intravenous piperacillin-tazobactam (4.5 g 3 times a day) plus oral doxycycline (100 mg twice a day) were initiated. Later that day the patient experienced respiratory failure and was transferred to the intensive care unit. During the days that followed, the patient was in a deep coma with decerebrate posture and multiorgan system failure. The skin rash became overtly petechial, with areas compatible with purpura fulminans. Because intravenous doxycycline is not available in Israel, doxycycline tablets were administered through the nasogastric tube, combined with intravenous meropenem. The patient died on October 2, 2005, on day 11 of illness (day 8 of hospitalization, day 5 of doxycycline therapy). An autopsy was performed and serum samples and tissue from various organs were preserved at –70°C for further study.

At autopsy, jaundice and edema with diffuse hemorrhagic rash, including the conjunctivae, were evident. Internal organs were congested, and moderate amounts of pleural fluid and ascites were noted. A pressure mark was evident on the left cerebellar tonsil, which indicated increased intracranial pressure. The cerebral cortex showed perivascular hemorrhages. Inflammatory cell infiltrates and occasional thrombi in the alveolar capillaries and arterioles were present in the lungs. Results of staining with silver-methenamine and periodic acid-Schiff were negative for pathogens.

**Figure 1:**
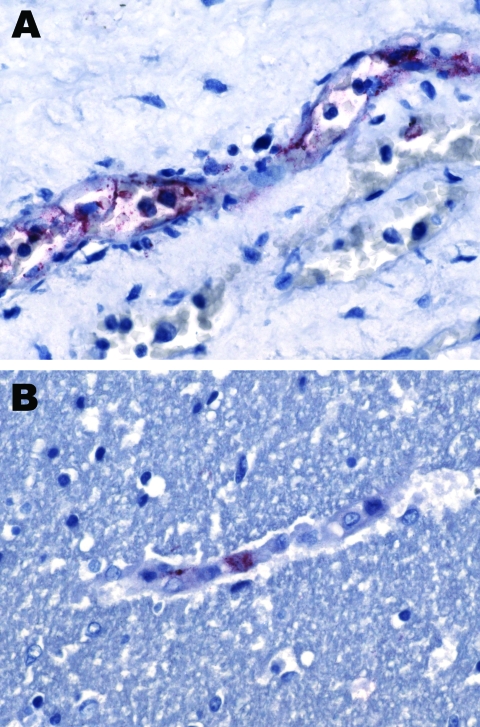
A small vessel in the kidney (A) and a capillary in the cerbral cortex (B) positive with immunohistochemical stain specific for spotted fever group rickettsiae**.** Original magnification ×158.

Immunohistochemical staining performed at the Centers for Disease Control and Prevention (Atlanta, GA, USA) (*3*) showed spotted fever group rickettsiae in the vascular endothelial cells of the patient’s brain and kidney (Figure 1). Serologic tests for *R. conorii* from day 7 of illness yielded negative results (both immunoglobulin [Ig] M and IgG). On day 11 of illness, IgG results remained negative and IgM results were borderline positive (Table).

**Table:** Diagnostic tests performed to identify spotted fever in the patient*

Day after disease onset	Assay	Specimen(s) tested	Result	Laboratory
7	IFA PCR	Serum Serum	IgM<100, IgG<100 Negative	IIBR† IIBR
11 (autopsy)	IFA	Serum	IgM = 64, IgG<32	CDC‡
	PCR	Serum sediment Liver, muscle, skin, lung, kidney Liver, muscle, skin	*Rickettsia conorii* subsp. *israelensis* Spotted fever group rickettsiae *R. conorii* subsp. *israelensis*	CDC IIBR§ CDC¶
	IHC stain#	Brain, kidney	Positive	CDC
	Cell culture**	Liver, lung	Negative	IIBR

*IFA, immunofluorescent assay; Ig, immunoglobulin; IIBR, Institute for Biological Research; CDC, Centers for Disease Control and Prevention; IHC, immunohistochemical. †IFA performed (*6*). Cutoff values for IgM and IgG are 100. ‡IFA performed (*7*). Cutoff values for IgM and IgG are 64. §Nested PCR for 17-kDa protein gene (*8*). ¶Nested and semi-nested PCR for 17 kDa protein gene and recombinant outer membrane protein A gene fragment, respectively, followed by sequencing (*9*). #Three-micron sections cut from formalin-fixed, paraffin-embedded brain and kidney tissue samples were stained by using an immunoalkaline phosphatase technique with a hyperimmune rabbit anti–*Rickettsia rickettsii* antibody at a dilution of <500 (*3*). **Homogenized samples from lung and liver were applied by centrifugation onto monolayer of Vero cells culture in 24-well plates and incubated for 2 weeks at 35°C in 5% CO_2_ atmosphere incubator.

**Figure 2:**
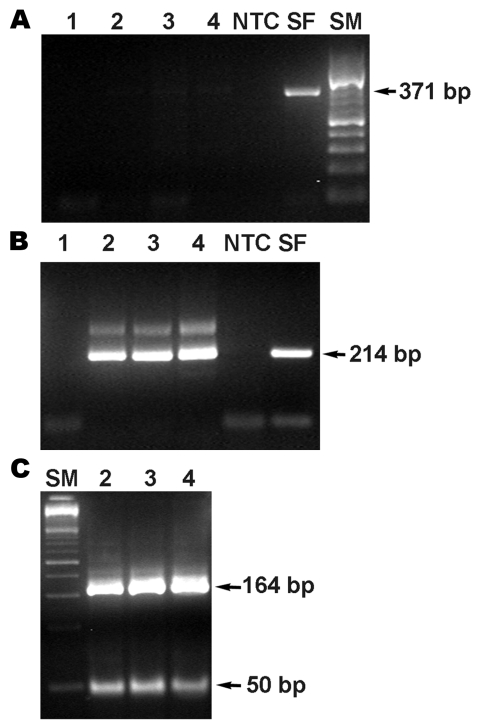
PCR product from the 17-kDa protein antigen gene obtained from DNA extracted from necropsied tissues of the patient. Primary PCR (A), nested PCR (B), and BfaI restriction enzyme pattern of the 17-kDa protein gene amplicon (C). Lane 1, reagent control; 2, skin; 3, liver; 4, lung; NTC, nontemplate control; SF, *Rickettsia conorii* DNA control; SM, size markers.

Results of nested PCR tests for spotted fever group rickettsiae (SFGR), performed at the Israeli National Reference Laboratory for Rickettsial Diseases on DNA samples prepared from serum collected on day 7 of illness (*8*), were negative. These tests were also applied to autopsy tissue samples (liver, muscle, skin, lung, kidney) and yielded a 214-bp amplicon from the 17-kDa protein gene of the SFGR (Figure 2). *Bfa*I restriction profile of 17-kDa protein gene amplicons consisted of 2 fragments, 50 and 164 bp that were identical to that of *R*. *conorii* subsp*. conorii* and *R. conorii* subsp*. israelensis* (*8*). A 208-bp fragment of the conserved 17 kDa *Rickettsia* spp. antigen gene was amplified at CDC from a DNA specimen obtained from a serum sample collected during the autopsy, and indicated SFGR DNA in the patient’s bloodstream. An outer membrane protein A (ompA) gene fragment (70–602 nt) was amplified from the positive serum sample extracted at CDC and from skin, liver, and muscle samples extracted at the Israel Institute for Biological Research as described (*9*). Nucleotide sequences of each of the 4 *ompA* amplicons (GenBank accession no. EU122392) and *R. conorii* subsp. *israelensis* (U43797, http://www.ncbi.nlm.nih.gov/entrez/viewer.fcgi?db=nuccore&id=1174108) were identical.

## Conclusions

This case underscores the difficulties involved in establishing the diagnosis of ISF during the acute phase of the illness. It also emphasizes the importance of considering that returning travelers may have acquired the illness locally. Although rickettsial infections can be acquired by travelers to India (*10*), the long incubation time (1 month) and the positive diagnosis of the etiologic agent as *R. conorii* subsp. *israelensis* makes this possibility unlikely. An endemic ISF case due to dog ownership is the more likely scenario. On the other hand, physicians caring for travelers returning from Mediterranean countries such as Italy, Portugal, and Israel should be alert to the possibility of ISF in febrile patients. Absence of eschar in ISF may be an obstacle to the correct diagnosis as exemplified by a recent case of a UK traveler to Portugal (*11*).

Israeli spotted fever is endemic in Israel (*2*). National surveillance data have been available only since the early 1970s (*10*). The incidence ranged from 0.7 to 10.3/100,000 (20–370 annual cases) from 1971 through 1980; it has declined steadily since 1980, reaching a nadir of 0.29/100,000 (20 cases) in 2004. The highest annual incidence reported was among children <10 years of age (10.5/100,000), and the lowest among persons >65 years (2/100,000) (*12*). A strong seasonal pattern exists, with the highest incidence occurring between June and October (*12*).

The case-fatality rate in Israel from 1971 through 1998 ranged from 0% to 3.5%, with an average rate of 0.7% (*12*). This rate may be an underestimate because seronegative fatal cases may not have been routinely investigated. Several examples of postmortem diagnoses in seronegative patients have been reported. In 1993, Yagupsky and Wolach described 2 children, whose postmortem diagnosis of ISF was established by using cell culture methods and animal inoculation studies (*5*). In 1997, 2 cases of unexplained deaths in young adults (31 and 38 years of age) were diagnosed after immunohistochemical detection of rickettsial antigen in paraffin-embedded tissue obtained at autopsy (*4**,**12*). Finally, nested PCR applied to sera and tissue in several serologically unconfirmed fatal cases of *R. conorii* infections was shown to be effective in establishing the correct diagnosis (*8**,**13**,**14*). PCR performed on whole blood or skin biopsy specimens of rash collected before treatment offers the possibility of improved early and rapid laboratory diagnosis of ISF and other rickettsial infections (*8**,**14**,**15*).
